# Kidney Elastography in Adult Nephrology: A Narrative Review

**DOI:** 10.3390/diagnostics16172732

**Published:** 2026-08-26

**Authors:** Nino Vreča, Nejc Piko, Sebastjan Bevc, Maša Knehtl

**Affiliations:** 1 Department of Nephrology, Clinic for Internal Medicine, University Medical Centre Maribor, Ljubljanska Ulica 5, 2000 Maribor, Slovenia; 2 Department of Dialysis, Clinic for Internal Medicine, University Medical Centre Maribor, Ljubljanska Ulica 5, 2000 Maribor, Slovenia; 3 Faculty of Medicine, University of Maribor, Ljubljanska Ulica 5, 2000 Maribor, Slovenia

**Keywords:** chronic kidney disease, kidney elastography, renal ultrasound, ultrasound elastography, shear wave elastography, renal fibrosis, kidney biopsy, non-invasive diagnostics, biochemical markers

## Abstract

Chronic kidney disease (CKD) represents a rising global health crisis traditionally monitored through routine biochemical markers that may not fully reflect underlying structural alterations. While renal biopsy remains the invasive reference standard for histopathological assessment, ultrasound elastography, particularly shear wave elastography (SWE), has emerged as a promising non-invasive imaging technique for assessing the mechanical properties of the renal parenchyma. This narrative review examines the role of renal elastography in adult nephrology, linking the pathophysiology of renal fibrosis with advances in clinical imaging. We discuss the biological mechanisms underlying changes in renal tissue biomechanics and review the available evidence regarding the association between elastography-derived stiffness measurements and histopathological fibrosis in native kidneys and renal allografts. Although several studies have demonstrated promising diagnostic potential, the clinical interpretation of renal stiffness remains challenging due to tissue anisotropy, renal perfusion and congestion, inflammation, obstruction, hydration status, body habitus, and acquisition-related factors. Current evidence supports renal elastography primarily as a potential adj method to conventional clinical, biochemical, and imaging assessment rather than as a replacement for kidney biopsy. Future integration of elastography with artificial intelligence, multiparametric ultrasound, and molecular biomarkers may further improve non-invasive renal phenotyping, although multicentre and longitudinal validation are required before widespread clinical use.

## 1. Introduction

CKD represents a rising global health crisis characterised by high morbidity, mortality, and economic burden. CKD is projected to become the fifth leading cause of death within the next two decades [1]. The rising global burden of kidney diseases necessitates continuous innovation in diagnostic and monitoring strategies. Currently, clinical management and monitoring of CKD relies primarily on the evaluation of serum creatinine-based estimated glomerular filtration rate (eGFR) and the quantification of urinary albumin excretion. These measures remain fundamental for diagnosis, risk stratification, and longitudinal monitoring; however, they provide predominantly functional rather than structural information. Conventional functional biomarkers such as serum creatinine and eGFR do not directly characterise renal tissue structure or histopathological changes, which may occur across the course of CKD [1,2]. Renal fibrosis is a common final pathway in the progression of CKD and is critical in determining long-term renal outcomes. Renal biopsy remains the reference standard for direct histopathological assessment of renal parenchymal injury and fibrosis; however, its invasive nature, sampling limitations, and procedural risks limit its suitability for repeated assessments. Ultrasound elastography comprises a group of techniques that assess mechanical properties by measuring tissue deformation or the propagation of mechanically induced shear waves. It was initially developed and validated in other organs, particularly the liver; elastography techniques have increasingly been investigated for renal assessment [3]. SWE has emerged as a promising, non-invasive imaging technique for quantitatively assessing renal tissue mechanical properties, most commonly through measurements of shear-wave speed (SWS) and derived elasticity parameters [4]. This narrative review article examines the current and emerging role of elastography in modern nephrology, linking the biological basis of renal fibrosis with clinical imaging. We discuss the pathophysiological mechanisms that drive biomechanical alterations in the renal parenchyma and review the available evidence regarding the association between elastography-derived stiffness measurements and biopsy-proven fibrosis in both native kidneys and renal allografts. Furthermore, we examine the biomechanical and methodological challenges currently limiting the routine use of elastography. Finally, we explore the potential future applications of renal elastography, including its integration with artificial intelligence (AI), multiparametric ultrasound, and dynamic prognostic modelling.

## 2. Pathophysiology of Renal Fibrosis and Biochemical Alterations

Renal fibrosis represents the common final pathway of progressive CKD, independent of the initiating aetiology [5]. It is a highly complex and dynamic process driven by interconnected molecular pathways. It arises from a dysregulated wound-healing response in which persistent injury prevents restoration of normal renal architecture [1,6]. Acute or sustained damage to the renal parenchyma, such as ischemia, metabolic toxicity, oxidative stress and chronic inflammation, leads to persistent disruption of the architecture of the kidneys [7]. Rather than resolving through tissue repair and epithelial regeneration, the sustained injury triggers a persistent inflammatory state [6]. This consequently leads to the progressive replacement of functional parenchyma with excessive and disorganised extracellular matrix (ECM), ultimately leading to nephron loss, compromised microvascular perfusion, and end-stage kidney disease (ESKD) [5,8].

Renal fibrogenesis is driven by complex interactions between injured tubular and endothelial cells, inflammatory cells, resident fibroblasts, pericytes, and multiple profibrotic signalling pathways. Sustained tissue injury promotes inflammatory responses and the release of profibrotic mediators, including transforming growth factor-beta (TGF-β) [9]. TGF-β and related signalling pathways prompt the activation of fibroblasts and pericytes and their differentiation toward myofibroblast phenotypes, which produce excessive amounts of ECM components, including fibrillar collagens and fibronectin [5,9]. Progressive ECM accumulation and remodelling alter the biomechanical properties within the tubulointerstitial compartment, contributing to increased tissue stiffness and reduced tissue compliance [9,10].

In recent years, advances in single-cell transcriptomics have further demonstrated that renal fibrosis is a heterogeneous and dynamic process. The progression of CKD is no longer viewed as a simple accumulation of collagen, but as a complex interplay between damage to the tubular epithelial cells, activated myofibroblasts, and infiltrating immune cells [5,11]. 

Studies published by Li et al. in 2022 and by Schuster et al. in 2023 have highlighted the role of distinct mesenchymal cell populations and the involvement of markers such as Platelet-Derived Growth Factor Receptor Beta (PDGFR-β) and Leucine-Rich Repeat Containing Protein 15 (LRRC15) in the fibrotic microenvironment [12,13]. This highlights the contribution of distinct mesenchymal cell populations to pathological ECM remodelling and renal fibrosis [12,13].

**Figure 1:**
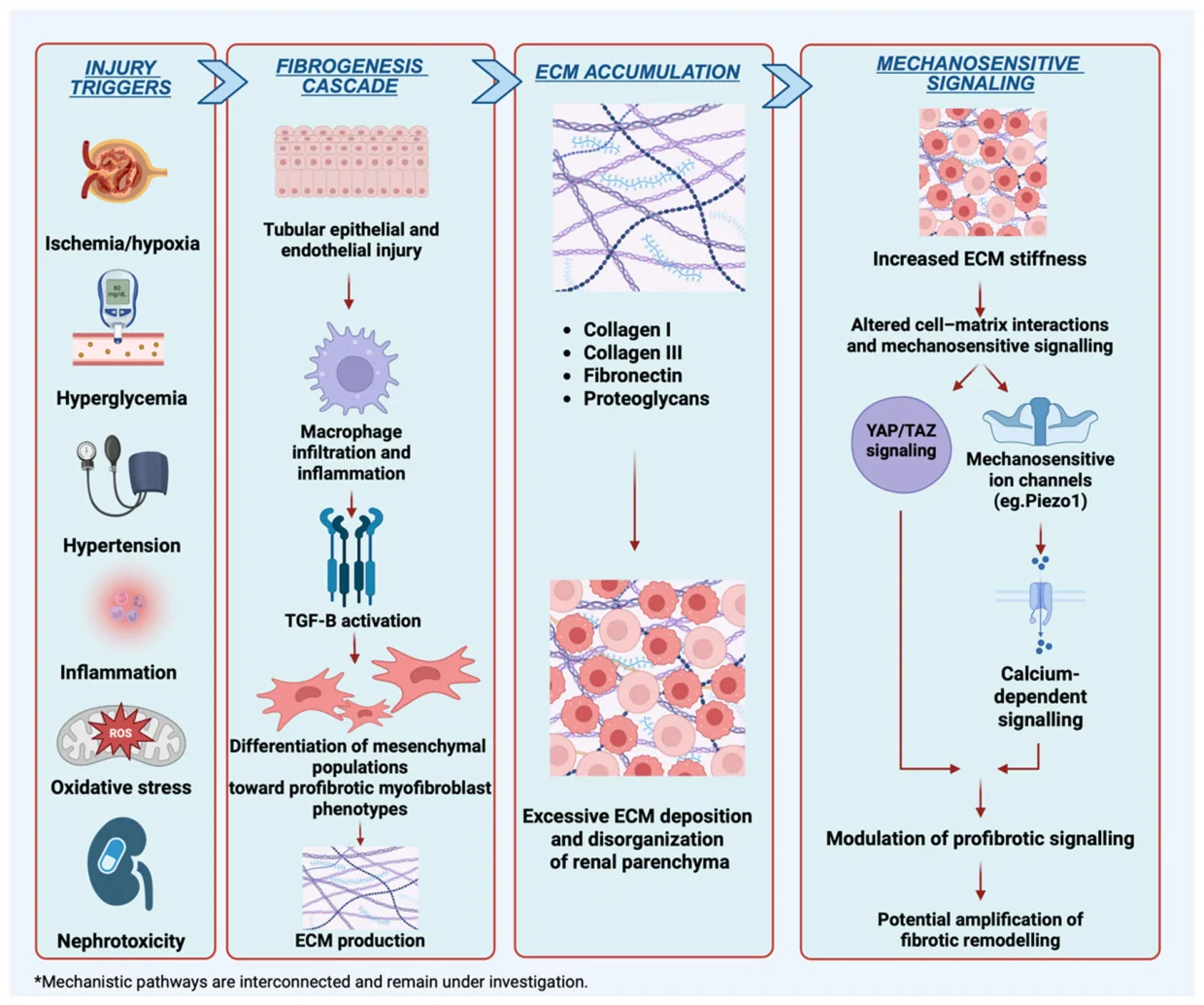
Pathophysiology of renal fibrosis and biomechanical alterations. Created by the authors based on [15,16,17,18].

Importantly, a discovery made in the last five years indicates that the ECM functions as a dynamic signalling hub rather than a passive structural scaffold [14]. In fibrotic tissue, increased matrix stiffness may alter cell–matrix interactions and activate mechanosensitive signalling pathways involving the Yes-associated protein (YAP) and transcriptional coactivator with PDZ-binding motif (TAZ), as well as mechanosensitive ion channels such as Piezo1 [15,16]. Experimental studies have demonstrated that mechanical stretch, compression, and increased matrix stiffness could activate Piezo1-mediated signalling in renal cells, promoting calcium-dependent profibrotic signalling [17]. Furthermore, inhibition of Piezo1 has attenuated renal fibrosis in experimental models, supporting a potential mechanobiological feedback between altered tissue mechanics and fibrotic remodelling [15,18]. The pathophysiology of renal fibrosis and biochemical alterations are shown in Figure 1.

The concept of elastography originates from the clinical use of tissue palpation to assess changes in tissue consistency [19]. However, the term “elastography” was first introduced into the medical lexicon by Ophir and colleagues in 1991 to define ultrasound-based techniques used for imaging tissue strain caused by applied compression. These early methods are nowadays known as strain elastography (SE). Further technological advancements led to the development of dynamic shear wave-based elastography techniques in which acoustic radiation force is used to generate shear waves within tissues and measure their propagation speed to estimate tissue mechanical properties. Magnetic resonance elastography (MRE) extended this concept by combining magnetic resonance imaging with externally induced mechanical vibrations to create spatial maps of tissue mechanical properties [19,20].

Initial widespread clinical use of elastography started predominantly in hepatology, where transient elastography (TE) (FibroScan^®^) revolutionised the non-invasive assessment of liver fibrosis. The method proved successful in diagnosing liver disease and determining fibrosis, which encouraged further exploration and adaptation of elastography for other organs, including the kidneys. However, renal elastography has not achieved the same level of widespread clinical use, partly due to the complex anatomical and functional characteristics of the kidneys, their retroperitoneal location, and their high vascular perfusion [19,21].

## 3. Ultrasound Elastography

Ultrasound elastography includes several methods that use ultrasound to assess the mechanical properties of different tissues. These can broadly be divided into strain-based techniques, which assess tissue deformation in response to an applied or physiological stress, and shear wave-based techniques, which generate and track mechanically induced shear waves to obtain quantitative measures of tissue mechanical properties. In nephrology, both approaches have been investigated, while shear wave-based techniques have become increasingly prominent in renal applications due to their ability to provide quantitative measurements without requiring manual compression [22].

### 3.1. Strain Elastography

SE, also known as compression elastography, was one of the pioneering elastography techniques. It measures tissue deformation, otherwise known as strain, in response to an internal or external mechanical stress. Stress can be applied manually with the ultrasound probe, or it can be caused by physiological movements such as cardiac pulsations or respiratory motion. For a given applied stress, softer tissues undergo greater deformation, therefore experiencing higher strain under the same amount of applied stress [23].

SE generally provides qualitative or semi-quantitative information. Elastographic findings are displayed as a colour-coded map on conventional ultrasound’s B-mode image, while quantitative assessment may be obtained using a strain ratio (SR) [24]. SR compares the observed target tissue to another reference tissue, usually subcutaneous fat or adjacent normal parenchyma. This is done by placing two regions of interest (ROI) in the observed and reference tissue. The SR is then calculated by comparing the strain of the reference tissue to the strain of the observed tissue. A higher SR generally indicates greater relative stiffness of the target tissue [24,25].

In clinical practice, SE has been investigated for renal fibrosis assessment, although its application to native kidneys is technically challenging. Static elastography methods using manual compression may be difficult to standardise due to the kidney’s positioning and the non-uniform pattern of fibrosis in many diffuse pathologies. However, a study conducted by Okda et al. reported that SE-derived SR values correlated positively with the histological degree of renal fibrosis and serum creatinine and negatively with eGFR [25].

### 3.2. Transient Elastography (Fibroscan^®^)

TE, commercially known as FibroScan^®^, uses a probe with an integrated piston-like vibrator to generate shear waves in the underlying tissue. The propagation and velocity of these shear waves are measured along a single ultrasound A-line by tracking the tissue displacement over time. The FibroScan^®^ system then calculates and reports a stiffness value in kPa [26].

TE is a widely used and validated non-invasive method for assessing liver fibrosis. Its application to native kidneys is limited due to the kidney’s position and depth, potentially interfering with ribs, bowel and their movements. When using TE for assessing kidney fibrosis, we must consider measurement failure in obese patients or in patients with ascites. Nevertheless, TE has been investigated in assessing renal allografts, which are typically located more superficially in the iliac fossa [27].

## 4. Shear Wave Elastography and Its Principles

SWE techniques are dynamic ultrasound-based techniques that provide quantitative information about tissue mechanical properties. Measurements are typically expressed as shear wave speed (SWS) or shear wave velocity (SWV), in metres per second (m/s). Some systems convert SWS into an elasticity-related parameter expressed in kilopascals (kPa), such as Young’s modulus (E). SWE is based on the generation of shear waves within the tissue and the subsequent tracking of their propagation [28].

While conventional B-mode ultrasound relies primarily on the reflection of longitudinal acoustic waves to create anatomical images, SWE additionally uses mechanically induced shear waves to characterise tissue mechanical properties [29]. SWE, particularly 2D-SWE, has been increasingly investigated for renal assessment as its real-time elastographic map may facilitate ROI placement and assessment of tissue heterogeneity [30].

SWE utilises a focused acoustic radiation force impulse (ARFI) generated by the transducer. This induces a minor micron-scale displacement in the tissue, generating shear waves that propagate transversely through the surrounding tissue. The ultrasound system then captures thousands of frames per second (plane-wave imaging) and tracks the propagation of the shear waves [31,32].

SWS represents the propagation speed of the generated shear waves and is related to the tissue’s shear modulus (G) [22,32]. In a simplified model of a homogeneous, isotropic, linear elastic, and nearly incompressible material, the Young’s modulus can be approximated from the shear modulus according to E ≈ 3G. However, biological tissues such as the kidney are heterogeneous and anisotropic, and these assumptions are not fully satisfied in vivo. Therefore, Young’s modulus values derived from SWS should be interpreted as model-dependent estimates rather than direct measurements of an intrinsic tissue property. In general, higher SWS corresponds to a higher estimated shear modulus and, under the assumptions of the applied model, greater tissue stiffness [29,32].

### 4.1. Point Shear Wave Elastography

pSWE uses a short, high-intensity acoustic pulse from the ultrasound transducer to transiently displace tissue within a small ROI. This approach is commonly based on acoustic radiation force impulse (ARFI) excitation. The generated shear waves propagate away from the excitation zone, and the same transducer is used to track their propagation and determine the SWV. Measurements are typically reported in m/s, although some systems provide converted values in kPa [29,33].

pSWE provides a quantitative measurement from a relatively small ROI. Multiple measurements are usually acquired from the tissue, and the median or mean value is reported. A limitation of pSWE is its lack of a real-time colour-coded elastographic map, which may help with identifying heterogeneous areas, large vessels, or artefacts and may contribute to greater operator variability [30,34].

A study conducted in 2016 by Cygan et al. demonstrated that reliable ARFI measurements require relatively homogeneous tissue and that the location and size of the measurement window can substantially influence pSWE results when regions with different tissue properties are included [35].

### 4.2. Two-Dimensional Shear Wave Elastography

2D-SWE techniques are based on generating shear waves across a larger 2D field of view, allowing the creation of a real-time, colour-coded quantitative elastogram shown on the conventional B-mode ultrasound image [28,34].

The visual stiffness map allows the operator to assess the spatial distribution of stiffness and more reliably place measurement ROIs within relatively homogeneous regions. A wider field of view also helps in avoiding structures such as large vessels or cysts, potentially improving measurement reproducibility. The size and location of the measurement ROI is generally adjusted according to the anatomical region being assessed, allowing targeted sampling of structures such as the renal cortex or medulla. Some systems also provide quality indicators, known as stability indexes, to further optimise data acquisition and lower the chance of user error [34,36].

## 5. Magnetic Resonance Elastography

MRE is a non-invasive imaging technique that combines the principles of elastography with the capabilities of magnetic resonance imaging to quantitatively map the stiffness of soft tissues in vivo [37].

The process of MRE starts with shear wave induction, where low-frequency mechanical vibrations are introduced into the target tissue using an external electromechanical driver system. Specialised motion-sensitive MR imaging sequences are used to capture the propagating shear waves within the tissue. These sequences encode the microscopic cyclic displacements caused by the shear waves into the phase of the MR signal, allowing for visualisation of the wave patterns within the tissue. Mathematical inversion algorithms analyse the measured wave fields to estimate tissue mechanical properties, which may include the shear modulus. The results are displayed as quantitative, colour-coded elastograms showing the spatial distribution of the measured mechanical properties. An advanced approach known as multifrequency MRE (mMRE) involves acquiring wave fields at multiple vibration frequencies rather than a single frequency and can provide additional information regarding the frequency-dependent mechanical behaviour of the tissue [38,39,40].

An important advantage of MRE is its ability to assess relatively deep organs without some of the depth limitations associated with ultrasound. It is also capable of providing volumetric maps of tissue mechanical properties. However, it requires specialised MRI hardware and external vibration equipment, is more expensive, and has longer acquisition times, which limits its accessibility and routine clinical application. Specific challenges for renal MRE include respiratory and physiological motion of the kidneys and the significant influence of renal perfusion and hydration status on measured values [40,41,42]. The main differences in elastography techniques are summarised in Table 1.

## 6. Challenges and Limitations of Kidney Elastography

Despite the potential of kidney elastography to provide non-invasive information on renal tissue mechanical properties, its widespread clinical use remains limited by several biomechanical, physiological, and methodological challenges. Unlike the liver, the kidneys are highly vascularized, anatomically complex, and dynamically influenced by changes in perfusion, pressure, and hydration. Furthermore, renal tissue exhibits marked structural heterogeneity and anisotropy, while patient-specific factors such as body habitus, kidney depth, and respiratory motion can affect measurement feasibility and reproducibility [43].

### 6.1. Anisotropy and Complex Renal Microarchitecture

One of the primary biophysical challenges in renal elastography is the intrinsic anisotropy of the kidney. Anisotropy refers to the dependence of measured mechanical properties on the direction of mechanical wave propagation relative to the underlying tissue architecture. The kidneys have a highly organised radial architecture comprising the vascular tree, loops of Henle, collecting ducts, and medullary pyramids, which contributes to direction-dependent mechanical behaviour. SWS measurements may vary according to the orientation of the ultrasound probe relative to the structures observed. Shear-wave measurements may vary according to the orientation of shear-wave propagation relative to the medullary pyramids [44]. In an in vivo porcine model, Gennisson et al. demonstrated significant differences in renal cortical and medullary elasticity measurements according to the orientation of the ultrasound probe relative to the main axis of the renal pyramids, providing experimental evidence of renal anisotropy [45]. In addition, the cortex, medulla, and renal sinus have distinct structural compositions and mechanical properties demonstrating different SWE-derived elasticity values, making the precise anatomical location of the ROI an important determinant of measured renal elasticity [46,47]. Accordingly, a recent clinical study conducted by Kuttancheri et al. in 2023 has used standardised cortical ROI placement while avoiding the medulla and vascular structures, highlighting the importance of consistent ROI selection and acquisition protocols for improving measurement reliability [34].

### 6.2. Hemodynamic and Physiologic Confounders

One of the most important limitations of renal elastography is the influence of renal haemodynamic and physiological factors on measured tissue mechanical properties. Renal blood flow (RBF) comprises roughly 20% of the total cardiac output, which is roughly 1 litre per minute. RBF is proportional to the difference in pressures between the renal artery and vein, but inversely proportional to the vascular resistance [48]. A recent comprehensive review by Giangregorio et al. published in 2026 highlighted that elevated central venous pressure and renal venous congestion may increase renal interstitial and vascular pressures and potentially alter measured stiffness. Following successful decongestion, organ stiffness measurements may decrease accordingly, indicating that elastographic measurements can reflect dynamic haemodynamic changes in addition to chronic structural alterations [49]. Acute clinical scenarios may further affect SWE measurements. Acute kidney injury can produce substantial changes in renal microcirculation and tissue, potentially altering elastographic measurements independently of established fibrosis [50]. Acute urinary tract obstruction may increase intratubular pressure, leading to false elevations in measured values [45]. Renal interstitial inflammation may also influence elastography-derived mechanical parameters, further complicating the interpretation of renal stiffness measurements [51]. These findings emphasise that elevated renal stiffness should not automatically be interpreted as evidence of fibrosis and that elastography should be interpreted alongside clinical, biochemical, Doppler, and conventional ultrasound findings.

### 6.3. Patient-Dependent Factors and Depth Limitations

Patient-related factors such as habitus and compliance represent an additional source of variability in renal elastography. Native kidneys are located deep within the retroperitoneum, and increasing distance from skin to the kidneys may attenuate the propagation of shear waves. Body habitus may also influence measured SWS independently of renal fibrosis, further complicating the interpretation of absolute stiffness values. A study on paediatric and adolescent patients, published in 2024 by Varda et al., showed that renal stiffness values were significantly higher in overweight patients and correlated strongly with fat mass; however, the study population was exclusively paediatric; therefore, these findings cannot be directly extrapolated to adults [52]. Respiratory motion represents another important technical limitation. The kidneys move substantially during respiration; therefore, SWE measurements are generally performed during breath-holding or controlled respiration to minimise motion-related variability and improve measurement reliability [3,53].

## 7. Kidney Biopsy and Kidney Elastography

The kidney biopsy remains the reference standard for histopathological assessment of many kidney diseases. Since its introduction, many advancements have been made in biopsy techniques to improve diagnostic value while minimising complications [54]. It serves as the established procedure for fibrosis assessment; however, its invasive nature, sampling limitations, and associated procedural risks limit its suitability for repeated assessment and longitudinal monitoring [55]. Renal biopsy provides a direct high-resolution assessment of the renal parenchyma, offering information for establishing an etiologic diagnosis, identifying active inflammatory processes, and grading the chronicity of structural damage, particularly interstitial fibrosis and tubular atrophy [56].

In recent years, multiple comparative studies have investigated SWE as a non-invasive method for assessing renal structural changes and fibrosis. A study conducted in 2020 by Leong et al. evaluated 75 patients with CKD undergoing renal biopsy and conventional SWE. The study demonstrated significant correlations between SWE-derived Young’s modulus and both the tubulointerstitial and the glomerular score. Young’s modulus also increased proportionally with the percentage of interstitial fibrosis and tubular atrophy, with an optimal cut-off Young’s modulus value of ≥5.81 kPa [57].

Islamoglu et al. investigated the relationship between elastography and pathologically identified interstitial fibrosis in native kidney biopsies. Their study included 57 patients, of whom only six had biopsy-detected fibrosis. Tissue stiffness was significantly higher in fibrotic kidneys than in non-fibrotic kidneys, with mean stiffness values of 12.83 ± 9.80 kPa and 7.76 ± 5.16 kPa. Elastography showed a negative predictive value of 91%, indicating that the method may have potential as an exclusion test for clinically significant fibrosis. However, sensitivity was 50%, specificity 62%, and positive predictive value 13%, and elastography measurements were not significantly correlated with the total histological score. It is also important to note that the small number of patients with fibrosis limits the strength of these findings [58].

Chen et al. conducted a prospective study in 2024, evaluating the diagnostic performance of SWE in a cohort of 162 patients with CKD who underwent renal biopsy. While SWE alone showed moderate diagnostic performance for differentiating the degree of renal fibrosis, the integration of SWE with eGFR improved its diagnostic performance. The study concluded that an integrated approach combining eGFR with SWE measurements improved diagnostic performance in differentiating mild renal fibrosis from moderate-to-severe fibrosis [36]. Importantly, the relationship between histopathological fibrosis and renal stiffness is not consistently linear across studies. Leong et al. reported lower cortical stiffness in patients with more advanced CKD and declining eGFR, contrasting with studies in which higher stiffness was associated with greater histopathological fibrosis. Despite these results, this combined strategy may provide additional diagnostic information beyond SWE alone [29].

A systematic review and meta-analysis conducted by Mo et al. in 2022 [30] included 405 patients and reported a pooled sensitivity of 84% (95% CI: 80–87%) and specificity of 80% (95% CI: 76–84%) for SWE in the assessment of renal fibrosis. Although these findings support the diagnostic potential of SWE, the reported thresholds varied between studies, and further clinical validation is required before universal cut-off values can be established [30].

Another meta-analysis conducted by Cao et al. in 2023 [59] specifically evaluated the performance of SWE with biopsy-proven renal fibrosis. By collecting data from 14 independent studies encompassing 1394 patients, the meta-analysis demonstrated good diagnostic performance of SWE for mild and severe fibrosis, while performance for intermediate fibrosis was moderate. These findings support an association between SWE-derived stiffness and histopathological fibrosis; however, further methodological standardisation and validation is needed before SWE can be used as a stand-alone diagnostic method for renal fibrosis [59].

### SWE in Evaluating Renal Allografts and Chronic Renal Allograft Injury

The clinical need for non-invasive monitoring is perhaps most pronounced in the management of renal allografts. Chronic allograft injury and progressive IFTA are the leading causes of transplant failure. Protocol biopsies of renal allografts are routinely utilised to detect subclinical allograft rejection or chronic changes; however, the risks of protocol biopsies present the need for a non-invasive follow-up method [55].

Recent comparative studies have investigated the diagnostic utility of SWE on renal allografts. A comprehensive systematic review by Filipov et al., published in 2024, collected data from 16 independent studies to evaluate the diagnostic accuracy of SWE in transplanted patients. The analysis showed a moderate correlation between kidney stiffness measured by SWE and biopsy results, with substantial between-study heterogeneity. The authors concluded that there was insufficient evidence to support elastography over biopsy in the longitudinal management of kidney transplant patients [60].

A prospective observational study conducted in 2024 by Ruidas et al. directly correlated SWE measurements with histopathological findings. The study concluded that SWE may serve as a non-invasive imaging tool to evaluate the renal allograft affected by chronic renal allograft injury. The authors therefore suggested that SWE could complement the regular follow-up imaging protocol along with regular 2D ultrasound and Doppler imaging [61].

Furthermore, a study conducted by Wang et al. in 2025 [62] studied the integration of SWE into dynamic prognostic models, highlighting its contribution beyond simple disease staging. They included 396 kidney transplant recipients and utilised machine learning to correlate SWE features with long-term allograft survival. The study developed and validated a predictive model to estimate the risk of high eGFR decline or ESKD. The addition of SWE to laboratory data may be effective for risk prediction. Medullary tissue stiffness of >10 kPa was associated with a higher probability of allograft deterioration [62]. The data from the studies are presented in Table 2.

## 8. The Future Perspectives of Renal Elastography

### 8.1. Artificial Intelligence and Automated Interpretation

The integration of artificial intelligence (AI) and machine learning algorithms represents an emerging area of research in renal elastography. Current elastographic assessments remain dependent on acquisition technique and the operator’s experience. Recent advances in deep learning may present a potential to overcome these limitations through automated image analysis and pattern recognition.

A study published in 2025 by Ye et al. developed a deep-learning model based exclusively on 2D-SWE images from biopsy-validated CKD patients. The model significantly outperformed both experienced and less-experienced radiologists in distinguishing mild from moderate-to-severe renal fibrosis. These findings suggest that AI-assisted interpretation may improve diagnostic consistency and reduce observer-related variability. It is important to note that the model was developed and evaluated in a single study population; therefore, external validation across different centres, ultrasound systems, and patient populations is required before its clinical implementation [63].

Machine learning approaches are also being investigated for prognostic modelling. Wang et al. demonstrated that incorporating SWE-derived stiffness parameters into an interpretable machine-learning framework improved the prediction of long-term adverse outcomes in kidney transplant patients [62].

### 8.2. Multiparametric Ultrasound and Integrated Imaging Biomarkers

A major limitation of current renal elastography is its inability to successfully distinguish fibrosis-related stiffness from hemodynamic influences such as perfusion, vascular congestion, inflammation, and urinary obstruction. To address this challenge, multiparametric ultrasound approaches combining SWE with other imaging modalities have emerged as a potential strategy. Chen et al. published a study in 2025 integrating the use of SWE with grayscale and Doppler ultrasound, which allowed simultaneous assessment of renal stiffness and intrarenal haemodynamics. The results demonstrated improved diagnostic performance compared with individual imaging modalities, suggesting that multiparametric ultrasound may help distinguish fibrosis-related changes from other factors influencing renal stiffness. Future ultrasound platforms may provide a method useful for evaluating fibrosis, perfusion, congestion, and inflammatory activity; however, more studies and external validation are needed before such models can be incorporated into routine clinical practice [64].

### 8.3. Integration with Molecular and Circulating Biomarkers

While imaging methods have proved useful in fibrosis assessment, there has also been some evidence suggesting that combining elastographic measurements with circulating and urinary biomarkers may provide complementary information regarding renal injury and fibrogenesis.

Several biomarkers, including kidney injury molecule-1 (KIM-1), neutrophil gelatinase-associated lipocalin (NGAL), TGF-β, procollagen-derived peptides such as PIIINP and Pro-C3, and markers of extracellular matrix turnover, have been investigated as potential markers of renal injury, fibrogenesis, and histopathological fibrosis, although their clinical utility for directly quantifying renal fibrosis remains incompletely established [65,66].

**Figure 2:**
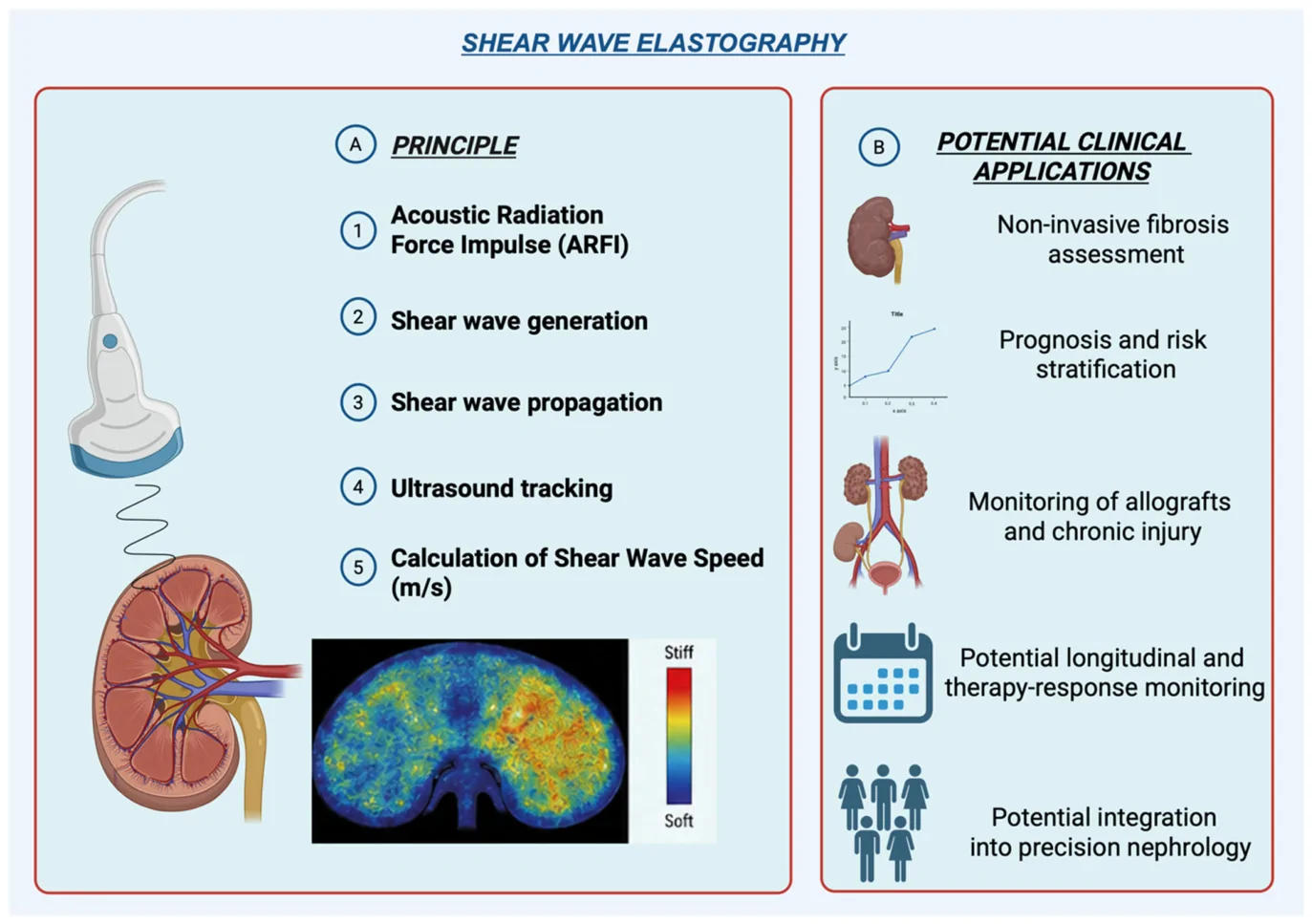
Principle and potential clinical applications of shear wave elastography.

A recent study published in 2025, conducted by Wang et al., combined SWE measurements with traditional renal function parameters such as serum creatinine and eGFR. The combined use of SWE and laboratory markers demonstrated higher diagnostic accuracy than models based on SWE or conventional renal function parameters alone [67]. Further examination and external validation are needed; however, these findings support the potential value of integrating elastography with established clinical and biochemical measures. Principles of SWE and its potential clinical applications are shown in Figure 2. 

## 9. Conclusions

Renal fibrosis remains a major pathological determinant of CKD progression. Advancements in molecular nephrology have transformed our understanding of fibrogenesis from a process of ECM accumulation into a dynamic network of inflammatory, cellular, and biomechanical interactions. The recognition that progressive tissue stiffening may contribute to fibrotic remodelling through mechanotransduction pathways, including YAP/TAZ signalling and Piezo1 activation, provides a biological rationale for investigating tissue stiffness as a potential biomarker of renal diseases.

Elastography has emerged as a promising non-invasive imaging approach for the assessment of renal tissue mechanical properties. Evidence from biopsy-correlated studies, systematic reviews, and meta-analyses indicates that SWE-derived stiffness measurements are associated with histopathological fibrosis and chronic structural changes in native kidneys and renal allografts. Furthermore, emerging studies suggest that elastographic parameters may provide prognostic information when integrated with conventional laboratory markers and clinical variables.

Nevertheless, important limitations remain. The complex biomechanical properties of the kidney, including tissue heterogeneity and anisotropy, together with the influence of perfusion, hydration, pressure, inflammation, obstruction, body habitus, and acquisition-related factors, complicate the interpretation and standardisation of renal stiffness measurements. Consequently, renal elastography should not currently be regarded as a direct or stand-alone diagnostic method for fibrosis, but rather as a complementary imaging biomarker that may provide additional information alongside clinical, biochemical, conventional ultrasound, and histopathological assessment.

Integration with AI, multiparametric ultrasound, molecular biomarkers, and prognostic modelling may further improve non-invasive renal phenotyping. However, these approaches remain investigational and require prospective validation before widespread clinical implementation. Overall, renal elastography shows promise as a complementary tool rather than a replacement for kidney biopsy. Its future clinical value will likely depend on its integration with other clinical, biochemical, imaging, and molecular approaches to provide a comprehensive assessment of renal structure, function, and disease progression.

## Figures and Tables

**Table 1:** Comparison of Elastography Techniques.

Technique	Strain Elastography (SE)	Point SWE (pSWE)	2D-SWE	Transient Elastography (TE)/FibroScan^®^	Magnetic Resonance Elastography (MRE)
**Principle**	Manual compression or tissue motion produces measurable tissue deformation	Focused acoustic radiation force generates localised shear waves	Multiple acoustic radiation excitations generate shear waves across a two-dimensional field of view	External mechanical vibrations generate shear waves	External mechanical vibrations generate shear waves
**Primary Output**	Colour-coded strain map with SR	SWS or SWV in m/s. Some systems provide elasticity-related values in kPa	Real-time, colour-coded elastogram; SWS/SWV or elasticity-related values depending on the system	Tissue stiffness-related measurements, typically reported in kPa	Quantitative maps of tissue mechanical properties
**Quantification**	Qualitative to semi-quantitative	Quantitative	Quantitative	Quantitative	Quantitative
**Advantages**	Widely available, simple concept, visual assessment of relative tissue deformation	Quantitative point measurement, no manual compression, small ROI	Larger field of view; visualisation of spatial heterogeneity, no manual compression	Rapid, well-validated for liver, can be used for superficial renal allografts	Volumetric assessment, relatively deep organs, not dependent on an ultrasound acoustic window
**Limitations**	Operator-dependent, difficult to standardise, difficult for deep organs	No global elastographic map, sampling error, ROI placement is crucial, affected by tissue depth and heterogeneity	Acquisition and ROI placement remain operator-dependent, affected by tissue depth and heterogeneity, anisotropy	Limited by tissue depth, affected by body habitus and acoustic access	High cost, limited accessibility, specialised equipment, motion artefacts

**Table 2:** Summary of principal biopsy-validated studies evaluating renal elastography.

Study	Population	n	Technique	Main findings
Leong et al.	CKD undergoing renal biopsy	75	SWE	SWE-derived Young’s modulus correlated with tubulointerstitial and glomerular scores and increased with the percentage of IFTA
Islamoglu et al.	Patients undergoing native kidney biopsy	57	SWE	Stiffness was higher in fibrotic than non-fibrotic kidneys (12.83 ± 9.80 vs. 7.76 ± 5.16 kPa)
Chen et al.	Patients with CKD undergoing renal biopsy	162	SWE	SWE showed moderate diagnostic performance; combining SWE with eGFR improved discrimination of mild versus moderate-to-severe fibrosis
Mo et al.	Biopsy-correlated studies included in meta-analysis	405	SWE	SWE demonstrated promising diagnostic performance for renal fibrosis
Cao et al.	Biopsy-correlated studies included in meta-analysis	1394	SWE	Good diagnostic performance for mild and severe fibrosis; moderate performance for intermediate fibrosis
Filipov et al.	Kidney transplant recipients	16	SWE	Moderate correlation between SWE-derived stiffness and biopsy findings
Ruidas et al.	Kidney transplant recipients	61	SWE	SWE showed potential for non-invasive assessment of chronic renal allograft injury
Wang et al.	Kidney transplant recipients	396	SWE	SWE features contributed to prediction of long-term allograft deterioration when integrated into a prognostic model

## Data Availability

No new data were created or analyzed in this study. Data sharing is not applicable to this article.
